# Regional metabolic heterogeneity of the hippocampus is nonuniformly impacted by age and caloric restriction

**DOI:** 10.1111/acel.12418

**Published:** 2015-11-02

**Authors:** Stephen A. Martin, Tyler M. DeMuth, Karl N. Miller, Thomas D. Pugh, Michael A. Polewski, Ricki J. Colman, Kevin W. Eliceiri, Timothy Mark Beasley, Sterling C. Johnson, Rozalyn M. Anderson

**Affiliations:** ^1^ Division of GeriatricsDepartment of MedicineSMPHUniversity of WisconsinMadisonWI53705USA; ^2^ Wisconsin National Primate Research CenterUniversity of WisconsinMadisonWI53715USA; ^3^ Laboratory for Optical and Computational InstrumentationUniversity of WisconsinMadisonWI53706USA; ^4^ Department of BiostatisticsUniversity of AlabamaBirminghamAL35294USA; ^5^ GRECCBirmingham/Atlanta Veterans Administration HospitalBirminghamAL35294USA; ^6^ GRECC William S. Middleton Memorial Veterans HospitalMadisonWI53705USA

**Keywords:** aging, metabolism, caloric restriction, hippocampus, mitochondria, neuroprotection

## Abstract

The hippocampus is critical for cognition and memory formation and is vulnerable to age‐related atrophy and loss of function. These phenotypes are attenuated by caloric restriction (CR), a dietary intervention that delays aging. Here, we show significant regional effects in hippocampal energy metabolism that are responsive to age and CR, implicating metabolic pathways in neuronal protection. *In situ* mitochondrial cytochrome c oxidase activity was region specific and lower in aged mice, and the impact of age was region specific. Multiphoton laser scanning microscopy revealed region‐ and age‐specific differences in nicotinamide adenine dinucleotide (NAD)‐derived metabolic cofactors. Age‐related changes in metabolic parameters were temporally separated, with early and late events in the metabolic response to age. There was a significant regional impact of age to lower levels of PGC‐1α, a master mitochondrial regulator. Rather than reversing the impact of age, CR induced a distinct metabolic state with decreased cytochrome c oxidase activity and increased levels of NAD(P)H. Levels of hippocampal PGC‐1α were lower with CR, as were levels of GSK3β, a key regulator of PGC‐1α turnover and activity. Regional distribution and colocalization of PGC‐1α and GSK3β in mouse hippocampus was similar in monkeys. Furthermore, the impact of CR to lower levels of both PGC‐1α and GSK3β was also conserved. The studies presented here establish the hippocampus as a highly varied metabolic environment, reveal cell‐type and regional specificity in the metabolic response to age and delayed aging by CR, and suggest that PGC‐1α and GSK3β play a role in implementing the neuroprotective program induced by CR.

## Introduction

Aging presents the greatest risk for neurodegenerative diseases and cognitive impairment. Caloric restriction (CR) without malnutrition delays aging and the onset of age‐related disease across diverse species, including mice and nonhuman primates (Anderson & Weindruch, 2010; Colman *et al*., 2014). Caloric restriction protects against age‐related neurodegeneration, loss of synaptic density, and cognitive impairment in mice (Fusco & Pani, 2013; Graff *et al*., 2013). Calorically restricted monkeys exhibit an attenuated brain aging phenotype (Colman *et al*., 2009), with preservation of gray and white matter volume, decreased brain inflammation and astrogliosis, and improved executive and motor function compared to *ad libitum* fed monkeys (Bendlin *et al*., 2011; Kastman *et al*., 2012; Sridharan *et al*., 2012, 2013).

Abnormalities in brain mitochondrial function are associated with Parkinson's disease, Alzheimer's disease, and Huntington's disease (Schon & Przedborski, 2011). The concurrence of mitochondrial dysfunction in these distinct neurodegenerative disorders suggests that mitochondrial efficiency is important in maintaining neural function and plasticity (Yin *et al*., 2014). Localized changes in brain energetics can act nonautonomously to influence metabolism in nearby regions; for example, in mice with cerebral cortex mosaic respiratory chain deficiency, progressive neurodegeneration is observed not only in the affected neurons but also in neighboring cells that are affected *in trans* (Dufour *et al*., 2008). More recent mouse studies have reported mitochondrial dysfunction in aged hippocampal neuronal progenitor cells (Stoll *et al*., 2011) and have identified mitochondrial integrity as a critical component in hippocampal long‐term potentiation and learning/memory (Pei *et al*., 2015). These observations underscore the importance of mitochondria and cellular energy metabolism in maintaining proper neuronal function.

Here, we investigated hippocampal energy metabolism using metabolic imaging of tissue sections to elucidate cell‐type and regional differences in metabolic status. We also determined the impact of aging on regional metabolic status and the chronology of change in metabolic parameters as a function of age. In adult mice, middle aged mice, and old mice, hippocampal mitochondrial cytochrome c oxidase, indices of nicotinamide adenine dinucleotide (NAD) metabolism, and levels and regional distribution of key mitochondrial regulator peroxisome proliferator‐activated receptor gamma coactivator 1 alpha (PGC‐1α) were measured *in situ* by quantitative imaging techniques. The impact of delayed aging by caloric restriction was determined on the above parameters in addition to mitochondrial succinate dehydrogenase activity and levels of the energy sensor AMPK (adenosine monophosphate‐activated protein kinase) to potentially identify changes that may be driving the brain aging process. To capture the potential for translatability of our findings, we also investigated hippocampal PGC‐1α and GSK3β levels and distribution in aged nonhuman primate rhesus macaque (*Macaca mulatta*) that were on control or CR diets (Colman *et al*., 2009, 2014). The goal of these investigations was to define hippocampal mitochondrial energy metabolism *in situ*, determine the impact of aging and delayed aging by CR, and investigate whether protection against age‐related functional decline is associated with changes in cellular energy metabolism.

## Results

### Temporal and regional specificity in age‐related changes in cellular energy metabolism

**Figure 1:**
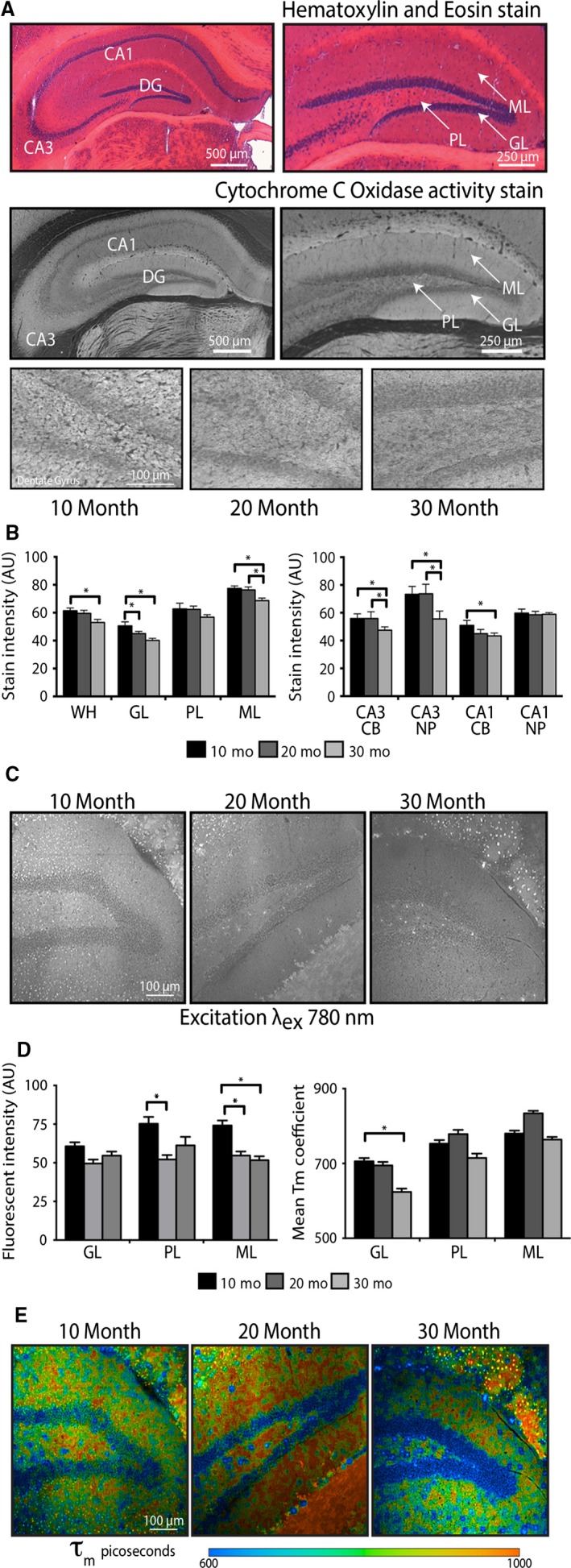
Aging induces a decline in mitochondrial activity in mouse hippocampus. (A) Reference hematoxylin and eosin and histochemical detection of cytochrome c oxidase activity (top & middle panels) in the indicated hippocampal regions. Representative cytochrome c oxidase activity stain images of DG (grayscaled and inverted). (B) Quantitation of hippocampal cytochrome c oxidase activity stain intensity for 10‐month‐old (*n* = 5), 20‐month‐old (*n* = 4), and 30‐month‐old (*n* = 4) mice. (C) Representative images showing fluorescence intensity of nicotinamide adenine dinucleotide (NAD)(P)H (_ex_λ_780_ nm) in the DG. (D) Quantification of NAD(P)H fluorescence intensity and mean fluorescence lifetime in the DG from 10‐month‐old (*n* = 5), 20‐month‐old (*n* = 4), and 30‐month‐old (*n* = 4) mice. (E) Representative images showing mean fluorescence lifetime (τ_m_) in picoseconds (_ex_λ_780_ nm). Data shown as average ± SEM (*P < 0.05). WH, whole hippocampus; DG, dentate gyrus; GL, granular layer; PL, polymorphic layer; ML, molecular layer; CB, cell bodies; NP, neuropil.

A major obstacle in defining the metabolic state in the brain is heterogeneity of the composite cellular population. The architecture of the hippocampus is complex, and highly specialized cells colocalize in discrete regions. Of particular importance is the dentate gyrus (DG), which is the primary site of neurogenesis in the adult brain and highly sensitive to age‐related atrophy. The DG is organized into three distinct layers: granule cells aligned along the granular layer (GL), a heterogenous neuronal and glial population in the polymorphic layer (PL), and axons and dendrites from the granule cells (neuropil) that populate the molecular layer (ML). To investigate the impact of aging on energy metabolism, we quantified maximal activity of cytochrome c oxidase (Complex IV), the terminal electron donor in the mitochondrial electron transport system (ETS), in whole brain 10‐μm cryosections from mice (Fig. 1A). Cytochrome c oxidase activity stain intensity was highly region specific within the hippocampus. In the inverted figure shown, greater stain intensity is depicted as brighter pixels. Quantitative analysis revealed significant differences among hippocampal regions (region effect: *P* < 0.001), a significant decline in cytochrome c oxidase activity with age (age effect: *P* < 0.05)‐ and region‐specific differences in the timing and degree of activity loss (age × region interaction: *P* < 0.001) (Fig. 1B). Post hoc analyses revealed significantly lower cytochrome c oxidase activity in the granular layer (GL) by 20 months of age (*P* < 0.05). For the molecular layer (ML), CA3, and CA1 regions, significant differences were detected in mice of 30 months of age only (*P*‐values <0.01, <0.001, <0.05, respectively).

The cofactors nicotinamide adenine dinucleotide (NAD) and nicotinamide adenine dinucleotide phosphate (NADP) play central roles in energy metabolism and intermediary metabolism. Due to the innate autofluorescence of the nicotinamide ring, levels and chemical properties of the reduced forms, NADH and NADPH, can be monitored directly and nondestructively in individual cells or tissue sections using high‐resolution microscopy techniques. Multiphoton laser scanning microscopy (MPLSM) quantifies NAD(P)H autofluorescence intensity (Denk *et al*., 1990) and fluorescence lifetime imaging microscopy (FLIM) (Lakowicz *et al*., 1992) quantifies the kinetics of photon release from the fluorophores. Autofluorescence intensity levels were region specific (*P* < 0.01), and the impact of age was region specific (age × region interaction: *P* < 0.05) (Fig. 1C,D). Within specific DG regions, GL was significantly different from PL and ML in 10‐month‐old animals (*P* < 0.001) although this difference was lost with age. Ten‐month‐old animals exhibited significantly higher autofluorescence intensity in the PL and ML (*P* < 0.05 and *P* < 0.05, respectively) than 20‐month‐old animals, while the GL was unaffected by age.

**Figure 2:**
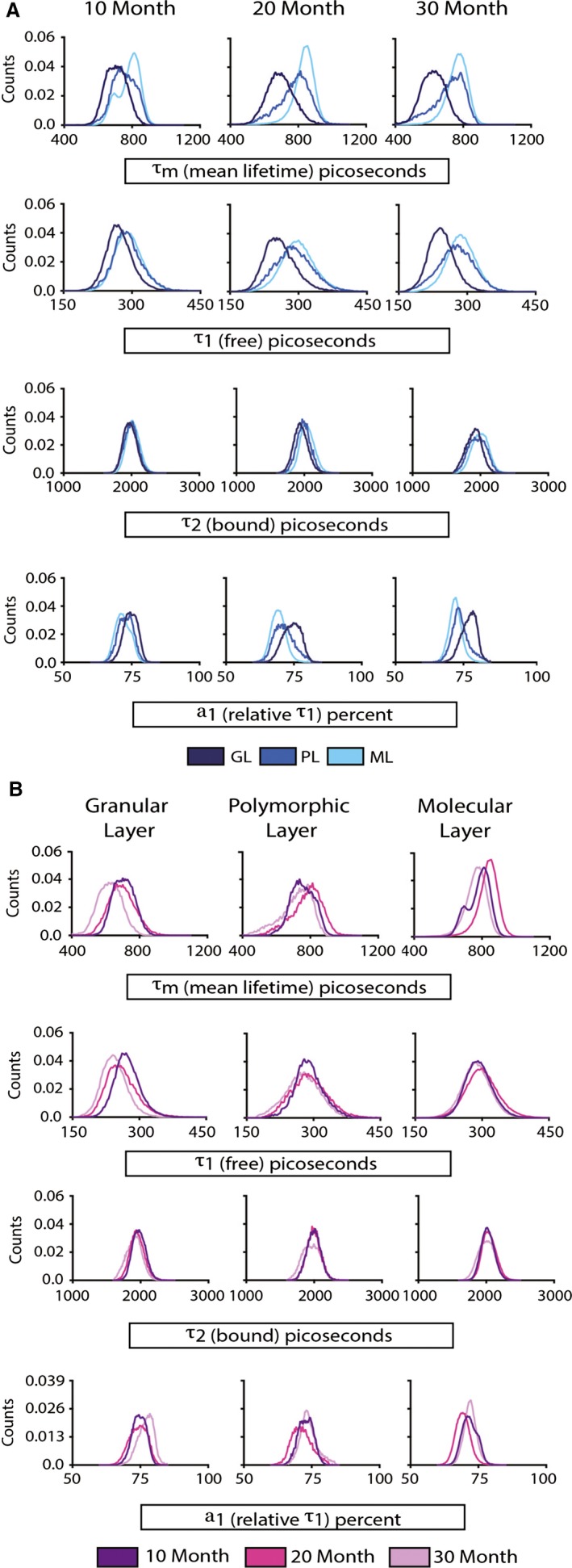
Aging impacts individual components of nicotinamide adenine dinucleotide (NAD)(P)H decay kinetics in the mouse dentate gyrus. (A) Distributions of mean fluorescence lifetime τ_m_ within the indicated hippocampal regions (top rows), fluorescence lifetime short component τ_1_ corresponding to free NAD(P)H (upper middle rows), long component τ_2_ corresponding to bound NAD(P)H (lower middle rows), and α_1_, the relative contribution of τ_1_ to τ_m_ (bottom row) are shown by region (A) and by age (B) for indicated regions from 10‐month‐old (*n* = 5), 20‐month‐old (*n* = 4), and 30‐month‐old (*n* = 4) mice (_ex_λ_780_ nm). GL, granular layer; PL, polymorphic layer; ML, molecular layer.

Fluorescence lifetime imaging microscopy detects changes in fluorescence lifetime, the duration that the NAD(P)H fluorophores stay in the excited state. For each specimen, the fluorescence decay curve was generated over multiple pulses and repeated for each pixel in the image capture field. The decay components can be quantified on a by‐pixel basis and color coding of decay in picoseconds allows differences among cell populations to be visualized. Combined over age groups, the mean lifetime (τ_m_) of NAD(P)H fluorescence significantly differed among DG regions (*P* < 0.001 for all comparisons of the GL, PL, and ML) (Figs 1E,F and 2). Additionally, among specific regions, the effect of age was not equivalent (age × region interaction *P* < 0.001). In the GL, the NAD(P)H decay kinetics of 30‐month‐old mice were significantly shorter compared to 10‐month‐old mice (*P* < 0.05). These data demonstrate that age impacts multiple metabolic parameters, that there is temporal separation of age‐related changes resulting in early and late events in the metabolic response to age, and that the impact of age is distinct among hippocampal regions.

### The metabolic microenvironment is distinct among hippocampal regions and the impact of age is region specific

We next looked at individual components that contribute to the τ_m_. The fluorescence lifetime curve fits as the sum of two exponentials, a short component τ_1_ and a long component τ_2_ that represent free and bound states of NAD(P)H, respectively, that contribute to the mean fluorescence lifetime as follows: τ_m_ = *a*_1_τ_1_ + *a*_2_τ_2_. For convenience, data are shown grouped by region (Fig. 2A) and by age (Fig. 2B). Decay values (τ_1_ and τ_2_) of NAD(P)H are influenced by the local cellular environment including redox, hypoxia, and pH, and in the case of τ_2_ the proteome to which the fluorophores are bound. Differences in the ratio of free/bound NAD(P)H are reflected in the *a*_1_, the relative contribution of τ_1_ to τ_m_ expressed as a percent. For example, cellular conditions favoring a glycolytic phenotype correlate with higher *a*_1_ values, that is, relatively increased contribution from free NAD(P)H (τ_1_), while an oxidative phenotype corresponds to lower *a*_1_ values, relatively increased ontribution from bound NAD(P)H (τ_2_) (Bird *et al*., 2005).

For the short lifetime component τ_1_, a significant main effect of region (*P* < 0.001) and an age × region interaction (*P* < 0.001) were detected (Fig. 2A,B, upper middle rows). Among regions, the GL, PL, and ML were all significantly different from each other (*P* < 0.001 for all comparisons). Post hoc analyses indicate that the differences among age groups were significant in the GL, where τ_1_ of 30‐month‐old mice was significantly lower compared to 10‐month‐old mice (*P* < 0.005). For the long lifetime component τ_2_, a significant main effect of region (*P* < 0.001) and age (*P* < 0.001), along with a significant age × region interaction (*P* < 0.001), was detected (Fig. 2A,B, lower middle rows). Combined over age groups, the τ_2_ significantly differed among DG regions (*P* < 0.001 for all comparisons of the GL, PL, and ML). Age × region interactions were observed in all DG regions. In the GL, all age groups significantly differed from each other. In the PL, 30‐month‐old mice exhibited significantly shorter τ_2_ compared to 10‐ and 20‐month‐old mice (*P* < 0.001 & *P* < 0.01, respectively). In the ML, 30‐month‐old mice exhibited significantly shorter τ_2_ compared to 20‐month‐old mice (*P* < 0.001).

The α_1_ value represents the relative contribution of τ_1_ to τ_m_ (Fig. 2A,B, bottom rows). Main effects of region (*P* < 0.001) and age were significant (*P* < 0.001) indicating regional differences in the proportion of free to bound NAD(P)H and a shift in these ratios with age. The significant interaction (*P* < 0.001) indicates that the effect of age was different across regions. Among regions, the GL, PL, and ML were all significantly different from each other (*P* < 0.001 for all comparisons). For GL, α_1_ in 30‐month‐old mice was significantly higher than that of 10‐ or 20‐month‐old mice (*P* < 0.001). For PL and ML, α_1_ was significantly lower in 20‐month‐old animals compared to 10‐ or 30‐month‐old animals (*P* < 0.001). Cumulatively, these data reveal region‐specific and age‐related differences in the chemical properties of free and bound NAD(P)H and in the ratios of free to bound NAD(P)H that are consistent with age‐related differences in cellular metabolism and in the cellular microenvironment.

### Hippocampal expression of mitochondrial regulator PGC‐1α is regionally distinct and altered with age

**Figure 3:**
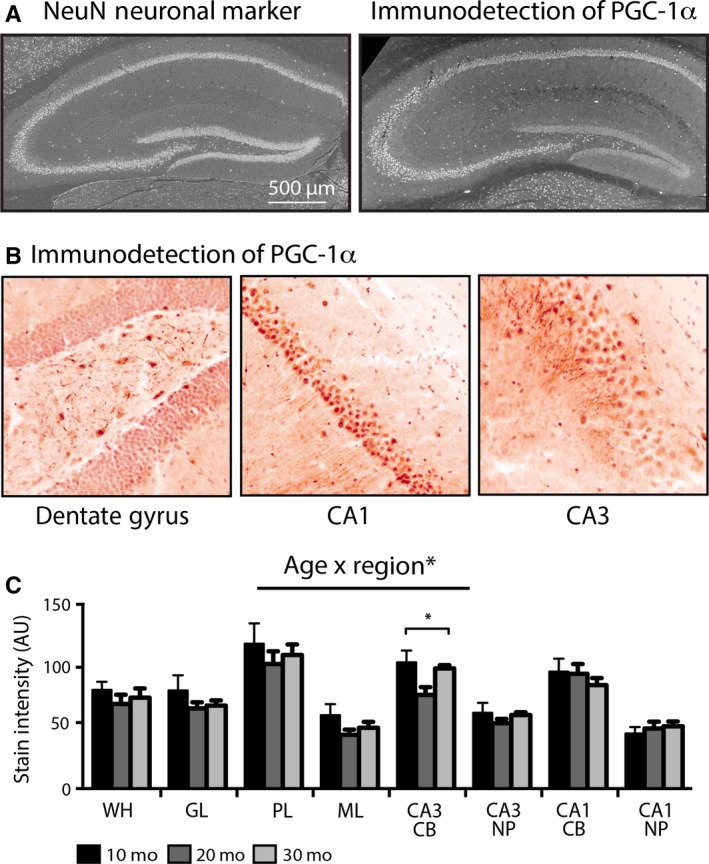
Regional differences in PGC‐1α protein expression and the impact of aging in mouse hippocampus. (A) Immunodetection of neuronal cell body marker NeuN (left) and PGC‐1α (right) in hippocampus. (B) High magnification of PGC‐1α in the indicated hippocampal regions from a 10‐month‐old mouse. (C) Quantitation of hippocampal PGC‐1α stain intensity of 10‐month‐old (*n* = 5), 20‐month‐old (*n* = 4), and 30‐month‐old (*n* = 4) mice. Data shown as average ± SEM (*P < 0.05). Age × Region* indicates significant interaction. WH, whole hippocampus; DG, dentate gyrus; GL, granular layer; PL, polymorphic layer; ML, molecular layer; CB, cell bodies; NP, neuropil.

PGC‐1α is a transcriptional coactivator that activates expression of both nuclear‐ and mitochondrial‐encoded ETS genes and has been implicated in aging and delayed aging by CR (Anderson & Prolla, 2009). PGC‐1α was detected throughout the hippocampus, and levels of expression were highest in neuronal populations as determined by staining for neuronal marker NeuN in adjacent sections (Fig. 3A). PGC‐1α localization to the cell bodies and the neuropil was apparent in DG, CA1, and CA3 (Fig. 3B). Quantitative analysis indicated that levels of PGC‐1α were significantly lower in the neuropil compared to adjacent cell bodies (*P* < 0.001) (Fig. 3C). A region main effect and an age × region interaction were both significant (*P* < 0.001). The lack of a main effect of age may be due to nonlinear changes in PGC‐1α, where absolute values for all regions except the CA1 were lowest in 20‐month‐old animals. In CA3 PGC‐1α, stain intensity was significantly lower at 20 than at 10 months of age (*P* < 0.05), but at 30 months of age, it was not different from that of 10‐month‐old mice. These data indicate that the age‐related differences in hippocampal metabolism in general are not explained by differences in levels of PGC‐1α protein, although this does not rule out the possibility for differences in PGC‐1α activity.

### The hippocampus undergoes metabolic reprogramming with caloric restriction

**Figure 4:**
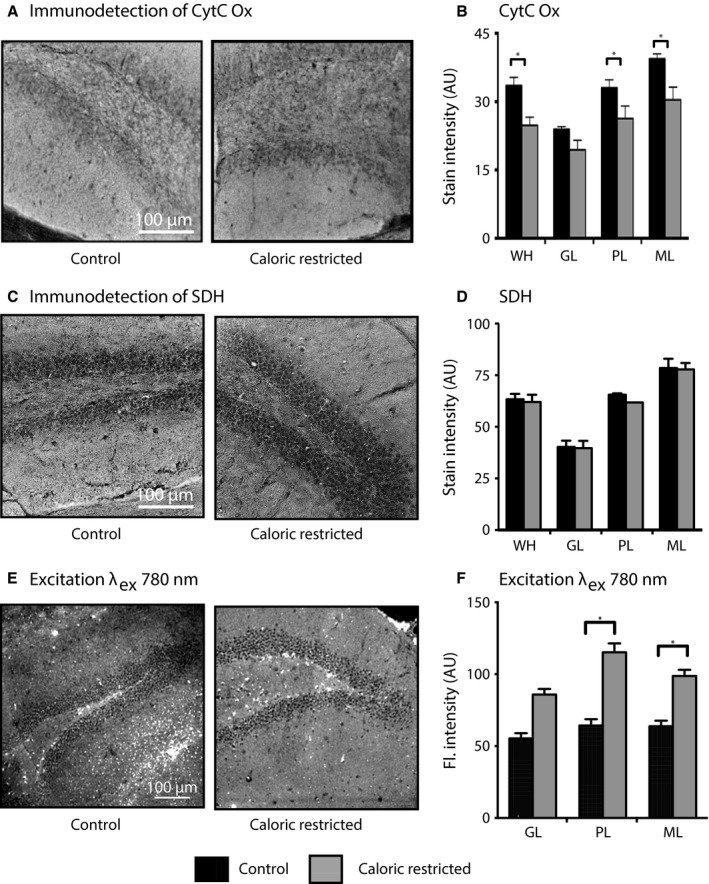
Caloric restriction induces a distinct metabolic state in mouse hippocampus. (A) Representative images of histochemical detection of cytochrome c oxidase activity in the DG from 20‐month‐old control and caloric restriction (CR) mice (grayscaled and inverted). (B) Quantitation of hippocampal cytochrome c oxidase stain intensity of control (*n* = 3) and CR mice (*n* = 3). (C) Representative images of histochemical detection of succinate dehydrogenase activity in the DG from 20‐month‐old control and CR mice (grayscaled and inverted). (D) Quantitation of hippocampal succinate dehydrogenase stain intensity of control (*n* = 5) and CR mice (*n* = 5). (E) Representative images showing fluorescence intensity of nicotinamide adenine dinucleotide (NAD)(P)H in the DG from 20‐month‐old control and CR mice. (F) Quantification of NAD(P)H fluorescence intensity (_ex_λ_780_ nm) in the GL, PL, and ML from control (*n* = 6) and CR (*n* = 6) mice. Data shown as average ± SEM (*P < 0.05). WH, whole hippocampus; DG, dentate gyrus; GL, granular layer; PL, polymorphic layer; ML, molecular layer.

The apparent decline in energy metabolism detected as a function of age and the ability of CR to delay brain aging prompted us to investigate the impact of long‐term CR (18 months intervention from 2 months of age) on metabolic parameters. Cytochrome c oxidase activity was measured in hippocampal sections from 20‐month‐old mice fed a control or CR diet (Fig. 4A). Unexpectedly, rather than opposing the age‐related decline in cytochrome c oxidase activity, stain intensity levels were lower in CR compared to controls (diet effect: *P* < 0.05; (Fig. 4B), and relative differences among regions were similar to those observed in controls (region effect: *P* < 0.001). Lower levels of activity of the Complex IV enzyme were significant for all regions of the DG except the GL (diet × region interaction for PL and ML: *P* < 0.05 & *P* < 0.005). Activity levels were also lower in the CB and NP of the CA3 region (*P* < 0.001) (Fig. S2). To understand whether this age‐related change in cytochrome c oxidase extended to other complexes of the ETS, we measured *in situ* activity of Complex II, succinate dehydrogenase. This enzyme catalyzes the oxidation of succinate to fumarate in the tricarboxylic acid cycle converting FAD to FADH_2_ and plays a role in anaplerosis. Although significant regional differences were detected for GL, PL, and ML (*P* < 0.005), mirroring the staining pattern for cytochrome c oxidase activity, there was no effect of CR arguing against a difference in mitochondrial density with CR. The key cellular energy sensor AMPK (adenosine monophosphate‐activated protein kinase) is activated by phosphorylation under conditions of energetic insufficiency (Burkewitz *et al*., 2014). Regional differences in hippocampal AMPK protein levels were detected (*P* < 0.001; all regions different except ML and CA3NP), but there was no effect of CR either on total AMPK levels or on phosphorylation status (Fig. S2), arguing against an energy deficit in CR hippocampus. Autofluorescence intensity of NAD(P)H measured using MPLSM was significantly higher in the DG of CR animals (diet main effect: *P* < 0.05, region × diet interaction: *P* < 0.005) (Fig. 4C,D). Within regions, significant increases were detected for the ML and PL (diet × region interaction for ML and PL: *P* < 0.05 & *P* < 0.005). Overall, absolute values of autofluorescence intensity in the DG were closely aligned with those of 10‐month‐old control fed mice suggesting a younger metabolic age. In contrast, the mean fluorescent lifetime τ_m_ was identical between control and CR in all hippocampal regions (Fig. S1). Modest differences were detected in τ_1_ for PL and GL (*P* < 0.05 for both), and a diet × region interaction was detected (*P* < 0.05) for *a*_1_, but a main effect of CR was not detected for any parameter. The impact of CR to increase autofluorescence intensity in the absence of appreciable changes in fluorescence lifetime is unexpected and likely presents a distinct metabolic state.

### Levels of hippocampal PGC‐1α and GSK3β are lower in animals on CR

**Figure 5:**
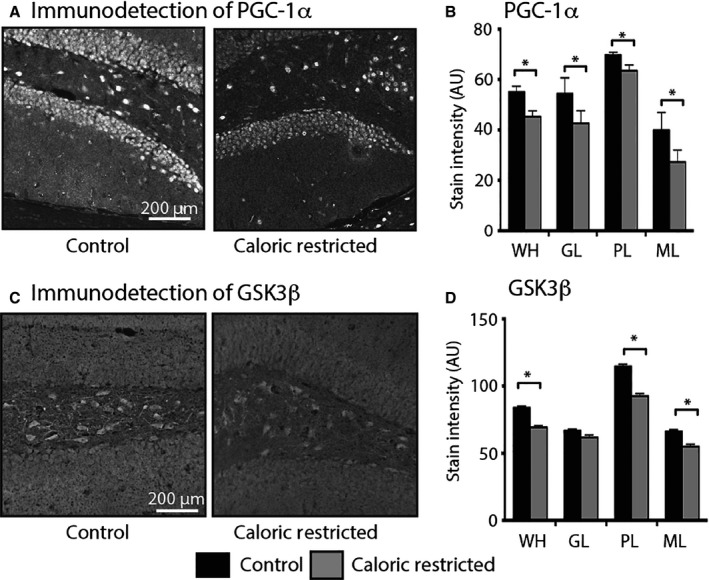
Impact of caloric restriction on expression of PGC‐1α and GSK3β in mouse hippocampus. (A) Representative images of PGC‐1α immunodetection in the dentate gyrus from 20‐month‐old control and CR mice (grayscaled and inverted). (B) Quantitation of PGC‐1α stain intensity in hippocampal regions from 20‐month‐old control (*n* = 3) and CR (*n* = 3). (C) Representative images of GSK3β immunodetection in the DG from 20‐month‐old control and caloric restriction (CR) mice. (D) Quantitation of GSK3β stain intensity indicated regions from 20‐month‐old control (*n* = 4) and CR mice (*n* = 5). Data shown as average ± SEM (*P < 0.05). WH, whole hippocampus; DG, dentate gyrus; GL, granular layer; PL, polymorphic layer; ML, molecular layer.

To understand the basis for CR's effects on metabolism, we measured protein levels and distribution of PGC‐1α (Fig. 5A). Regional differences in PGC‐1α expression were similar to those observed in control animals (region effect: *P* < 0.001). PGC‐1α levels were significantly lower in hippocampi from CR mice (diet effect: *P* < 0.05) (Fig. 5B). Within regions, the impact of CR on PGC‐1α levels was significant for the GL (*P* < 0.001), PL (*P* < 0.05), and ML (*P* < 0.001) of the DG and in the pyramidal cells of the CA3 (*P* < 0.001). The ability of PGC‐1α to activate expression of mitochondrial genes is regulated in part by GSK3β (Anderson *et al*., 2008), a nutrient‐sensitive kinase strongly implicated in the etiology of Alzheimer's disease (Sereno *et al*., 2009; DaRocha‐Souto *et al*., 2012). Like PGC‐1α, GSK3β is enriched in neurons and levels are regionally specific (*P* < 0.001) (Fig. 5C,D). Caloric restriction significantly influenced total GSK3β protein levels (diet effect: *P* < 0.05) in a region‐specific manner (diet × region interaction: *P* < 0.001). Post hoc analysis revealed that the levels of GSK3β were significantly lower in CR than control fed mice for the ML (*P* < 0.05) and the PL (*P* < 0.001). GSK3β is regulated in part by inhibitory phosphorylation on serine 9 (Forde & Dale, 2007; Phukan *et al*., 2010). Phosphorylated GSK3β was sequestered in cell bodies and displayed region‐specific differences in levels (region effect: *P* < 0.001) that were influenced by CR to the extent that there was an interaction between diet and region (*P* < 0.001) (Fig. S2). The ratio of phospho‐GSK3β to total GSK3β tended to be higher in the DG with CR in mice, although this did not reach statistical significance.

**Figure 6:**
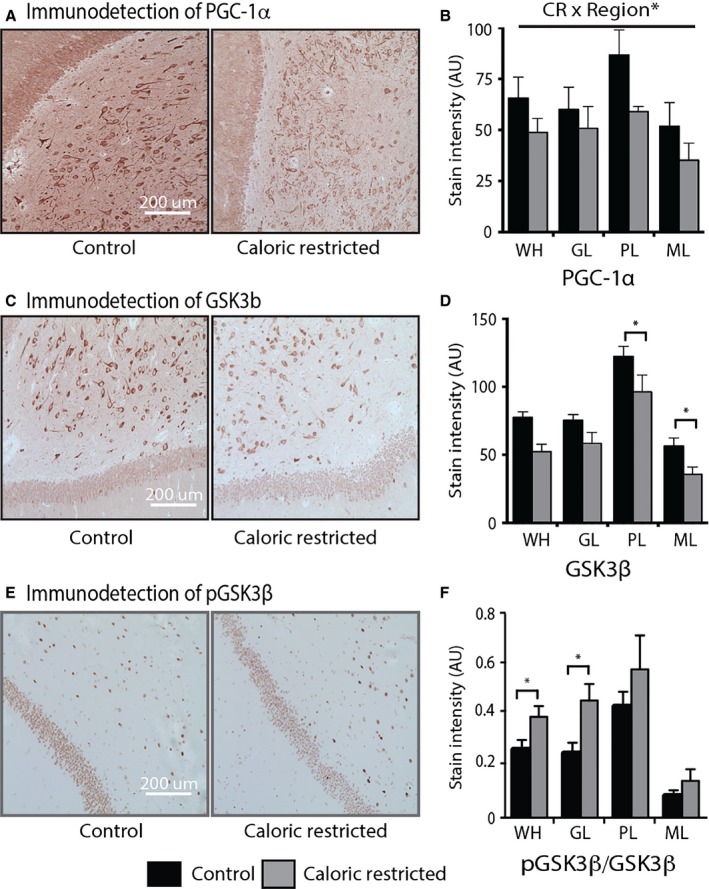
Impact of caloric restriction on expression of PGC‐1α and GSK3β in rhesus monkey hippocampus. (A) Representative images of PGC‐1α immunodetection in the DG from control (left; 23 years old) and caloric restriction (CR) (right; 18 years old) monkeys. (B) Quantitation of PGC‐1α stain intensity in the hippocampus of old control (*n* = 3) and CR monkeys (*n* = 5) (average 24 years of age, range 17–32 years of age). (C) Representative images of GSK3β immunodetection in the DG from control (23 years old) and CR (29 years old) monkeys. (D) Quantitation of GSK3β stain intensity in the hippocampus of control (*n* = 7) and CR monkeys (*n* = 6). (E) Representative image of pGSK3β immunodetection in the DG from control (25 years old) and CR (25 years old) monkeys. (F) Ratio of pGSK3β/total GSK3β in indicated regions from control (*n* = 7) and CR monkeys (*n* = 6). Data shown as average ± SEM (*P < 0.05). CR × Region* indicates significant interaction. WH, whole hippocampus; DG, dentate gyrus; GL, granular layer; PL, polymorphic layer; ML, molecular layer.

We next investigated whether the effect of CR in mice was similar in nonhuman primates. These experiments included fixed brain tissues from rhesus monkeys involved in the 25‐year ongoing Caloric Restriction and Aging study at the Wisconsin National Primate Research Center (Colman *et al*., 2009). Data were generated for monkeys in the age range of 15–32 years, which represents older middle age to advanced age, the average lifespan being ~26 years for rhesus monkeys in captivity. The CR diet was initiated in adulthood (~10 years of age). The beneficial effect of CR on brain aging has been previously reported for this cohort (Colman *et al*., 2009; Bendlin *et al*., 2011; Kastman *et al*., 2012; Sridharan *et al*., 2013). Hippocampal regional distribution of PGC‐1α protein in rhesus monkeys (Figs S3 and 6A) was similar to that detected in mice (Fig. 3). In rhesus monkeys, PGC‐1α levels were higher in the neurons of the PL and the pyramidal cells of the CA3, resulting in a significant regional effect (*P* < 0.001). Levels of PGC‐1α tended to be lower for each region in CR monkeys, and the diet × region interaction was significant (*P* < 0.001) (Fig. 6B). GSK3β protein levels and distribution were also similar between rhesus monkeys (Fig. 6C,D), and mice (Fig. 4). In monkeys, levels of total GSK3β were highest in the neurons of the PL, and differences among regions were significant (region effect: *P* < 0.001). Furthermore, GSK3β protein levels were lower in CR monkeys in the PL (*P* < 0.005) and ML (*P* < 0.05) (Fig. 6C), matching the impact of CR in mice. Levels of the serine 9 phosphorylated form of GSK3β significantly differed across hippocampal region (region effect: *P* < 0.001), but there was no effect of CR on levels of phospho‐GSK3β (Figs S4 and 6E). The ratio of phospho‐GSK3β to total GSK3β was significantly higher with CR in the GL (*P* < 0.05) and ML (*P* < 0.05) (Fig. 6F). As was the case in mice, GSK3β protein levels and distribution were closely aligned with that of PGC‐1α (Fig. 6A,D). These data demonstrate that the previously observed neuroprotective effects of CR in hippocampus may be linked to reduced levels and activity status of PGC‐1α and GSK3β.

## Discussion

We have used *in situ* measures to probe energy metabolism in the hippocampus and show that metabolic status is regionally distinct and that aging causes changes in hippocampal energy metabolism that are temporally separated and region specific. An age‐related decline in expression of nuclear‐encoded mitochondrial genes has previously been described in the fontal cortex in humans (Lu *et al*., 2004), and lower capacity for ATP synthesis has been reported in mitochondrial isolates from the basal ganglia of rhesus monkeys (Pandya *et al*., 2015). In the hippocampus in rats, age lowers expression of nuclear‐encoded ETS genes (Zeier *et al*., 2011). Our data in mice support these findings and further reveal regional and cell‐type specificity in metabolic status and in the impact of age and CR. As might be expected, cytochrome c oxidase activity was greatest in the mitochondria‐rich neuronal extensions of the ML. The impact of age in lowering cytochrome c oxidase activity in the ML occurred late in the aging process, whereas the impact of age on neuronal cell bodies in the GL occurred earlier at 20 months. It is not yet clear which aspects of neuronal function are most sensitive to loss of mitochondrial activity, or the extent to which subtle changes in energy efficiency contribute to age‐related functional decline.

Multiphoton laser scanning microscopy and FLIM complement mitochondrial activity studies allowing *in situ* quantitative analysis of NAD(P)H total levels and photon decay properties among adjacent cell populations. One of the most striking findings of our study is the distinctive difference in metabolic signatures within the DG and how age‐related changes are nonuniform. Neurons of the GL had the lowest autofluorescence intensity, highest *a*_1_, shortest τ_1_, and shortest τ_m_, and these data together with the low mitochondrial activity values suggest that this neuronal population is more glycolytic. With age, these parameters shifted unidirectionally, with significantly increased *a*_1_, decreased τ_1_, and shorter τ_m_, and even lower mitochondrial activity. The ML, in contrast, had higher autofluorescence intensity than GL, lower *a*_1_, longer τ_1_, longer τ_m_, and the highest mitochondrial activity values in the DG, suggestive of a more oxidative phenotype. Age induced a biphasic shift in *a*_1_, where the lowest value was detected at 20 months of age. In all three DG regions measured, aging was accompanied by a shortening of τ_2_ that has been interpreted as a lowering of the contribution of NAD(P)H to the decay curve (Blacker *et al*., 2014). Although there is insufficient evidence to define this age‐related change in τ_2_ in terms of metabolic flux, these data support the broader concept that metabolic shifts as a function of age are life‐phase specific.

The impact of CR in lowering cytochrome c oxidase activity was an unexpected outcome of this study. Increased expression of nuclear‐encoded mitochondrial genes is a hallmark of CR in multiple tissues, including regions of the brain (Barger *et al*., 2015). It is unlikely that lower cytochrome c oxidase reported here is an index of lower mitochondrial activity; the documented beneficial effect of CR in preserving cognitive function and delaying indices of hippocampal aging is unlikely to be conferred under conditions of energetic dysfunction. The SDH data suggest that the lower cytochrome c oxidase activity cannot be explained by a decline in mitochondrial number or mitochondrial function. *Ex vivo* studies using exogenous application of sera from mice on 6 months of CR to primary cerebellar neurons have been reported to result in increased cytochrome c oxidase accompanied by mitochondrial biogenesis (Cerqueira *et al*., 2012). The data shown here are not consistent with CR‐induced mitochondrial biogenesis in the hippocampus *in vivo*, as activity of SDH *in situ* was equivalent between control and CR animals. The key to the cytochrome c oxidase data may lie in the distinction between maximal activity and *in vivo* capacity, where maximal activity can be reduced without compromising energetic production under normal physiological conditions. The subunit composition of the ETS complexes is influenced by local factors including mitochondrial membrane lipid composition, abundance of reactive oxygen species, and whether glycolytic or oxidative metabolism is favored (Fukuda *et al*., 2007; Hagopian *et al*., 2010; Bartesaghi *et al*., 2015). These adaptive changes can influence maximal activities of the ETS without inducing defects in energy metabolism. The absence of difference between control and CR mice in hippocampal levels of the energy sensor AMPK, including total protein and phosphorylation/activation status, suggests that the lower cytochrome c oxidase activity detected in CR animals might be viewed as an adaptation to changes in the cellular environment rather than a loss of function *per se*.

The lack of overt differences in fluorescence lifetime measures between control and CR at 20 months of age was unexpected, but this may be a question of which stage of adulthood is being investigated. Fluorescence lifetime *a*_1_ values in the DG were shifted lower at 20 months of age in control mice, suggesting a greater reliance on oxidative metabolism in the DG in middle age. At the whole body level, mice on CR rely more on oxidative metabolism (Bruss *et al*., 2010) and short‐term studies have demonstrated increased circulating levels of ketone bodies (Mahoney *et al*., 2006), a key source for oxidative metabolism in the brain. It will be interesting to determine whether the previously described neuroprotective effects of ketone bodies (Maalouf *et al*., 2007) are mediated by adaptations in energy metabolism.

In mice and monkeys, CR results in lower levels of PGC‐1α and its companion regulator GSK3β. The precise role of the PGC‐1α pathway in regulating brain energy metabolism and neuronal health is unclear. PGC‐1α knockout induces brain lesions (Lin *et al*., 2004), and knock down negatively impacts dendritic growth and synaptic density in cultured neurons (Cheng *et al*., 2012), but it is less clear what impact more nuanced manipulations of PGC‐1α levels would have on brain energy metabolism and neuronal function. Regulation of PGC‐1α activity is highly complex and dependent on numerous post‐translational modifications. The NAD^+^‐dependent deacetylase SIRT1 positively regulates PGC‐1α through deacetylation and has been implicated in neuronal protection (Kim *et al*., 2007; Michan *et al*., 2010). It remains to be seen whether age impacts hippocampal SIRT1 activity or PGC‐1α acetylation status. GSK3β regulates PGC‐1α protein stability and gene target activation in non‐neuronal cells (Anderson *et al*., 2008). GSK3β has long been implicated in the etiology of Alzheimer's disease including neurofibrillary tangles (Sereno *et al*., 2009), amyloid‐β processing, and plaque formation (DaRocha‐Souto *et al*., 2012). Activation of GSK3β in the mouse forebrain reversibly induces tangles, astrocytosis, and learning deficiencies (Engel *et al*., 2006), while inhibition of GSK3β overcomes cognitive decline associated with diabetes (Datusalia & Sharma, 2014). GSK3β and PGC‐1α are colocalized in hippocampus of mice and monkeys, and in both species, CR lowers levels of GSK3β and PGC‐1α. Lower levels of GSK3β are predicted to slow turnover of PGC‐1α with possible consequences for cellular distribution and transcriptional co‐activation. We propose that the PGC‐1α response to changes in GSK3β function may be important in aging and neurodegenerative disease.

While we are still in the early stages in uncovering the role of metabolism in brain aging, our findings emphasize the importance of cell type, brain region, and life phase in the transition to old age. The accord in CR response between mice and monkeys suggests that findings of this study are likely relevant to human brain aging. The evidence presented herein is consistent with a role for metabolism in age‐related disease vulnerability and functional decline and suggests that metabolism itself may be a suitable target for preventative or corrective intervention.

## Experimental procedures

### Animals

All animal protocols were approved by the Institutional Animal Care and Use Committee at the University of Wisconsin, Madison.

### Mouse study

Six‐week‐old male B6C3F1 hybrid mice were obtained from Harlan Laboratories (Madison, WI, USA) and housed under controlled pathogen‐free conditions in accordance with the recommendations of the University of Wisconsin Institutional Animal Care and Use Committee. For the aging study, mice were fed 87 kcal week^−1^ of control diet (Bio‐Serv diet #F05312) from 2 months of age and were individually housed. This level of calorie intake is ~95% of *ad libitum* for the B6C3F1 strain so all food was consumed. By 30 months of age, mortality of the control animals was ~45%, consistent with the expected lifespan for this strain. Mice were euthanized by cervical dislocation at 10, 20, and 30 months of age. For the diet study, a parallel group of mice were fed a caloric restricted diet of 73 kcal week^−1^ from 2 months of age, which was a 16% reduction from control mice in the aging study (Bio‐Serv diet #F05314). The 20‐month‐old CR mice were 17% lower in body weight, 30% lower in fat mass, and 10% lower in lean mass than age‐matched control fed mice. In our hands, this dietary regimen results in 73% survival at 30 months of age. Brains were isolated, bisected, embedded in OCT, frozen in liquid nitrogen, and stored at −80 °C until further processing.

### Monkey study

Eighteen rhesus monkeys between 17 and 32 years of age (average age = 24.2 years at necropsy) were included in this study. Age at death was used as a covariate in all analyses to control for the range of ages. The animals were part of the Aging and Caloric Restriction longitudinal study conducted at the Wisconsin National Primate Research Center. Details of the CR manipulation, housing, and husbandry have been previously described (Ramsey *et al*., 2000; Colman *et al*., 2009). Briefly, control animals were fed *ad libitum,* and CR animals were fed 30% fewer calories relative to their own baseline *ad libitum* intake. The length of the calorie‐restricted diet ranged from 12 to 17 years and was initiated when the animals were 8–14 years of age. Upon death, brains were harvested, sectioned according to a standard necropsy protocol, fixed overnight in 10% neutral‐buffered formalin, and paraffin‐embedded.

### Multiphoton laser scanning microscopy

Autofluorescence detection and fluorescence lifetime imaging were conducted using the multiphoton workstation at the University of Wisconsin Laboratory for Optical and Computational Instrumentation (LOCI, www.loci.wisc.edu), and the system design and instrumentation have been previously described in detail (Pugh *et al*., 2013). Briefly, coronal cryostat sections of mouse hippocampus (10 μm) were placed on glass slides, dried, and mounted with glass coverslip with Clear‐Mount mounting solution (Electron Microscopy Sciences). The instrument response function of the optical system was calibrated before each imaging session (Bird *et al*., 2004). A Nikon CFI Plan Apo 20× lens (Melville, NY, USA) was used for all imaging. Data were collected using an excitation wavelength of 780 nm, and emission was filtered at 457 ± 50 nm, the spectral peak for NADH/NADPH. The data collection time was 240 s using a pixel frame size of 256 × 256. The system has multiple detectors including a 16 channel combined spectral lifetime detector (utilizes a Hamamatsu PML‐16 PMT), detection range 350 to 720 nm, and a H7422P GaAsP photon counting PMT (Hamamatsu) for intensity and lifetime imaging. Acquisition was performed with WiscScan, a LOCI developed acquisition package software. Autofluorescence intensity and fluorescence lifetime data were analyzed in SPCImage (Becker & Hickl, v.2.9.1, Berlin, Germany) where a Levenberg–Marquardt routine for nonlinear fitting was used to fit the fluorescence decay curve collected for each pixel in the 256 × 256 frame to a model multi‐exponential decay function. Data were assessed by the minimized chi‐square value generated during the fit so that analysis was unbiased. To eliminate background fluorescence a threshold for analysis was applied based on photon counts; contributions from lipofuscin, which emits photons with very low τ_m_, were excluded from the analysis. Data were binned at >1000 photons/pixel to maintain high quality of the fit. For autofluorescence intensity, regions of interest were described using 12 × 12 pixel boxes, measures included 10, 8, and 6 boxes per image for the molecular, granular, and polymorphic layers, respectively, and data were analyzed in ImageJ (NIH, Wayne Rasband, http://rsb.info.nih.gov/ij/). For fluorescence lifetime, regions of interest were defined using the inclusion tool in SPCImage, where concentric inclusion zones were defined and values for intermediate layers were calculated by subtraction of distributions.

### Histochemistry and immunodetection

Serial cryostat sections 10 μm in thickness were cut at −14 °C with a Leica Cryostat (Fisher Supply, Waltham, MA, USA), defrosted and air‐dried, and stained for cytochrome c oxidase and succinate dehydrogenase enzymatic activity as previously described (Pugh *et al*., 2013). For each experiment, tissues were sliced, batches were processed, and data were captured start to finish within 24 h. Immunodetection of PGC‐1α and GSK3β was conducted as previously described (Pugh *et al*., 2013) following antigen retrieval on 10‐μm cryosections (mouse) or 5 μm fixed (monkey) tissues. Antibodies and reagents used are as follows: biotinylated anti‐mouse IgG or biotinylated anti‐rabbit IgG (Vector Labs, Burlingame, CA, USA), peroxidase‐labeled avidin biotin complex (ABC) solution (Vector Labs), ImmPACT NovaRED reagent (Vector Labs), PGC‐1α (sc‐13067; Santa Cruz Biotechnology), AMPK (2532; Cell Signaling Technologies, Danvers, MA, USA), threonine 72 phospho‐AMPK (2535; Cell Signaling Technologies), total GSK3β (9315; Cell Signaling Technology), serine 9 phospho‐GSK3β (9336; Cell Signaling Technology), and NeuN (MAB377; Millipore, Billerica, MA, USA). With the exception of the multiphoton imaging, stained slides were imaged with a Leica (Buffalo Grove, IL, USA) DM4000B microscope and photographed with a Retiga 4000R digital camera (QImaging Systems, Surrey, BC, Canada). Camera settings were optimized for each stain; for uniformity, all images for a given stain were taken on the same day with identical settings, fixed light levels, and fixed shutter speed optimized at each magnification. Digital images were converted from color to monochrome and inverted, so that greater stain intensity is shown as brighter pixels. All image analysis was performed using Adobe Photoshop (Adobe Systems, San Jose, CA, USA). Stain intensity was measured using either the rectangular marquee tool in the hippocampal region of interest or the lasso outline tool. Within each region for each stain, the size of the capture box was uniform with an average inclusion of ~30K pixels. Cytochrome c oxidase measures included 6–14 individual boxes per region. In mice, immunodetection of PGC‐1α, GSK3β, and phospho‐GSK3β involved 8–10 individual boxes or lasso capture of 9–10 neuronal cell bodies for the polymorphic layer and 20–30 for CA1 and CA3 regions. In monkeys, PGC‐1α, GSK3β, and phospho‐GSK3β were measured using 6–10 individual boxes per region or lasso capture of 25–36 neuronal cell bodies per region. Background correction was conducted using an unstained adjacent area of the slide. Consistency among batches of specimens was confirmed using replicate samples where relative differences between regions and experimental groups were repeatedly detected and found to be equivalent.

### Statistical analysis

To account for the dependence among observations due to multiple measurements per animal, we performed linear mixed models (LMM) assuming a compound symmetric covariance structure using SAS PROC MIXED (Littell *et al*., 2006). The LMMs included full factorial with type 3 tests of the main effects and interactions. To explore the age‐by‐region interaction, simple main effects were investigated whether there were age effects within each region and whether there were region differences within each age group. To explore the CR‐by‐region interaction, simple main effects were investigated whether there were CR effects within each region. We employed no formal multiple testing correction. Instead, consistent with published guidelines for statistical reporting (Saville, 1990), exact *P*‐values are reported.

## Author contributions

SAM and TMD conducted the quantitative imaging analysis, KNM conducted the multiphoton analysis, TDP and MAP prepared the specimens and conducted the histochemistry and immunodetection, KWE consulted on the multiphoton imaging, RJC and SCJ consulted on the nonhuman primate work, TMB conducted the statistical analysis, RMA designed the study, SAM and RMA wrote the manuscript.

## Funding

This work was supported by NIH/NIA R01 grants AG037000, AG043125, and ADRC grant AG033514. SAM and KNM are supported by T32 fellowships from the UW Institute on Aging AG000213 and the UW Department of Nutritional Sciences DK007665, respectively. The study was conducted with the use of resources and facilities at the William S. Middleton Memorial Veterans Hospital, Madison, WI.

## Conflict of interest

None declared.

## Supporting information


**Fig. S1** Impact of CR on NAD(P)H decay kinetics in the mouse dentate gyrus.
**Fig. S2** Impact of caloric restriction on expression of cytochrome c oxidase activity, succinate dehydrogenase activity, AMPK, and pAMPK in mouse hippocampus.
**Fig. S3** Impact of caloric restriction on expression of PGC‐1α and GSK3® in mouse hippocampus.
**Fig. S4** Impact of caloric restriction on expression of pGSK3® in mouse hippocampus.
**Fig. S5** Representative image of immunodetection of hippocampal PGC‐1α in an 18‐year‐old rhesus monkey.
**Fig. S6** Impact of caloric restriction on expression of PGC‐1a, GSK3®, and pGSK3® in rhesus monkey hippocampus.
