# Supplementary material for: Regional metabolic heterogeneity of the hippocampus is nonuniformly impacted by age and caloric restriction
Source: Aging Cell. 2015 Nov 2;15(1):100–10. doi: 10.1111/acel.12418 (PMC4717265; doi:10.1111/acel.12418)
Supplement: Supplementary file 1 — Fig. S1 Impact of CR on NAD(P)H decay kinetics in the mouse dentate gyrus. Fig. S2 Impact of caloric restriction on expression of cytochrome c oxidase activity, succinate dehydrogenase activity, AMPK, and pAMPK in mouse hippocampus. Fig. S3 Impact of caloric restriction on expression of PGC‐1α and GSK3® in mouse hippocampus. Fig. S4 Impact of caloric restriction on expression of pGSK3® in mouse hippocampus. Fig. S5 Representative image of immunodetection of hippocampal PGC‐1α in an 18‐year‐old rhesus monkey. Fig. S6 Impact of caloric restriction on expression of PGC‐1a, GSK3®, and pGSK3® in rhesus monkey hippocampus. [file ACEL-15-100-s001.pdf]

Supporting Information

Supplementary Figure Legends

Fig.S1. Impact of CR on NAD(P)H decay kinetics in the mouse dentate gyrus. Distributions of the mean fluorescence lifetime τ_m (top row), the short component τ_1 corresponding to free NAD(P)H (upper middle row), long component τ_2 corresponding to bound NAD(P)H (lower middle row), and $a_{1,}$ the relative contribution of τ_1 to τ_m (bottom row) are shown by (A) region and (B) diet for the granular layer (GL), polymorphic layer (PL), molecular layer (ML) from 20-month-old Control (n=6) and CR (n=6) mice, ($_{ex}\lambda_{780}$nm).

Fig.S2. Impact of caloric restriction on expression of cytochrome c oxidase activity, succinate dehydrogenase activity, AMPK, and pAMPK in mouse hippocampus. (A) Quantification of cytochrome C oxidase stain intensity in the CA3 Cell Bodies (CA3CB) and CA3 Neuropil (CA3NP) from Control (n=3) and CR mice (n=3). (B) Quantification of succinate dehydrogenase stain intensity in the CA3 Cell Bodies (CA3CB) and CA3 Neuropil (CA3NP) from Control (n=5) and CR mice (n=5). (C) pAMPK stain intensity within the whole hippocampus (WH), granular layer (GL), polymorphic layer (PL), molecular layer (ML), CA3 Cell Bodies (CA3CB), and CA3 Neuropil (CA3NP) from Control (n=5) and CR mice (n=5). (D) pAMPK stain intensity within the whole hippocampus (WH), granular layer (GL), polymorphic layer (PL), molecular layer (ML), CA3 Cell Bodies (CA3CB), and CA3 Neuropil (CA3NP) from Control (n=3) and CR mice (n=3). (E) Representative images of AMPK immunodetection in the dentate gyrus from a Control mouse (grayscaled and inverted). Data shown as average ± SEM (*$p<0.05$)

Fig.S3 Impact of caloric restriction on expression of PGC-1α and GSK3β in mouse hippocampus. (A) Quantification of PGC-1α stain intensity in the CA3 Cell Bodies (CA3CB) and CA3 Neuropil (CA3NP) from Control (n=3) and CR mice (n=3). (B) Quantification of GSK3β stain intensity in the CA3 Cell Bodies (CA3CB) and CA3 Neuropil (CA3NP) from Control (n=4) and CR mice (n=5). Data shown as average ± SEM (*p<0.05)

Fig.S4 Impact of caloric restriction on expression of pGSK3β in mouse hippocampus. (A) Quantification of pGSK3β stain intensity within the whole hippocampus (WH), granular layer (GL), polymorphic layer (PL), molecular layer (ML), CA3 Cell Bodies (CA3CB), and CA3 Neuropil (CA3NP) from Control (n=4) and CR mice (n=5). (B) Representative image of pGSK3β immunodetection in the dentate gyrus from a Control mouse (grayscaled and inverted). (C) Quantification of pGSK3β/total GSK3β ratio within the whole hippocampus (WH), granular layer (GL), polymorphic layer (PL), molecular layer (ML), CA3 Cell Bodies (CA3CB), and CA3 Neuropil (CA3NP) from Control (n=4) and CR mice (n=5). Data shown as average ± SEM (*p<0.05). CR x Region* indicates significant interaction.

Fig.S5. Representative image of immunodetection of hippocampal PGC-1α in an 18-year-old rhesus monkey. PGC-1α is detected throughout the hippocampus.

Fig.S6. Impact of caloric restriction on expression of PGC-1a, GSK3β, and pGSK3β in rhesus monkey hippocampus. (A) Quantification of PGC-1α stain intensity in the CA3 Cell Bodies (CA3CB) and CA3 Neuropil (CA3NP) Control (n=7) and CR (n=6) monkeys. (B) Quantification of GSK3β stain intensity in the CA3 Cell Bodies (CA3CB) and CA3 Neuropil (CA3NP) Control (n=7) and CR (n=6) monkeys. (C) Quantification of pGSK3β

stain intensity within the whole hippocampus (WH), granular layer (GL), polymorphic layer (PL), molecular layer (ML), CA3 Cell Bodies (CA3CB), and CA3 Neuropil (CA3NP) from Control (n=7) and CR (n=6) monkeys. (D) Quantification of pGSK3β/GSK3β in the CA3 Cell Bodies (CA3CB) and CA3 Neuropil (CA3NP) Control (n=7) and CR (n=6) monkeys. Data shown as average ± SEM (*$p<0.05$)

Figure S1

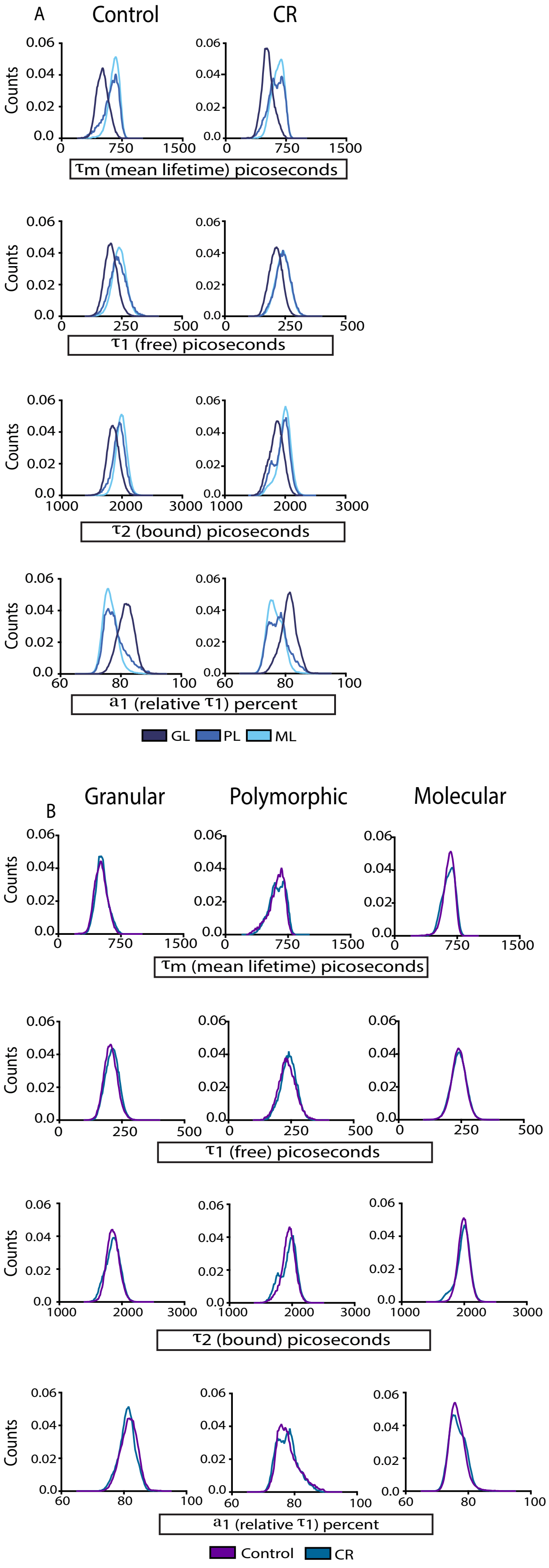
A
Control
CR
Counts
τm (mean lifetime) picoseconds
τ1 (free) picoseconds
τ2 (bound) picoseconds
a1 (relative τ1) percent
GL
PL
ML
B
Granular
Polymorphic
Molecular
Control
CR

Figure S2

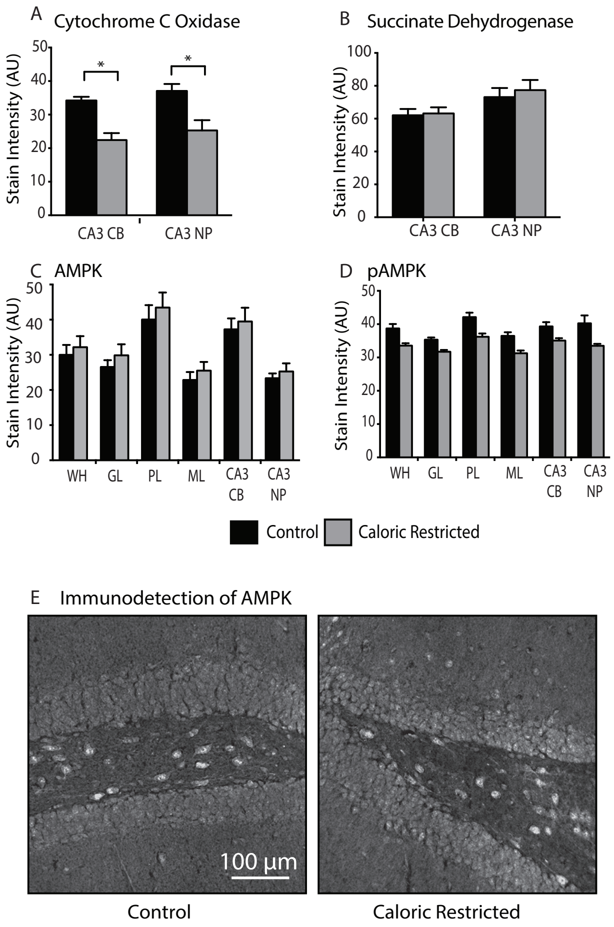

A Cytochrome C Oxidase
Stain Intensity (AU)
CA3 CB
CA3 NP
B Succinate Dehydrogenase
Stain Intensity (AU)
CA3 CB
CA3 NP
C AMPK
Stain Intensity (AU)
WH
GL
PL
ML
CA3 CB
CA3 NP
D pAMPK
Stain Intensity (AU)
WH
GL
PL
ML
CA3 CB
CA3 NP
Control
Caloric Restricted
E Immunodetection of AMPK
100 µm
Control
Caloric Restricted

Figure S3

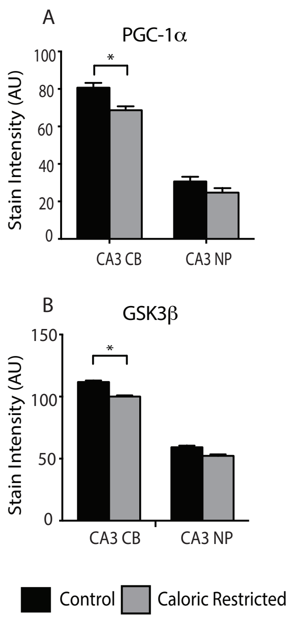

A
PGC-1α
100
80
60
40
20
0
*
Stain Intensity (AU)
CA3 CB
CA3 NP
B
GSK3β
150
100
50
0
*
Stain Intensity (AU)
CA3 CB
CA3 NP
Control
Caloric Restricted

Figure S4

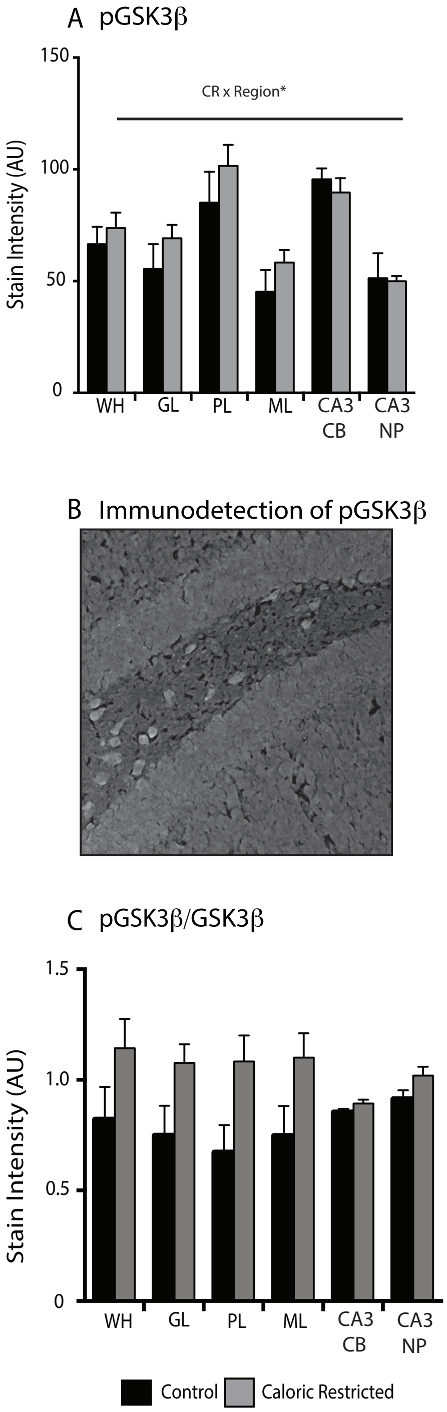
A pGSK3β
150
CR x Region*
100
50
0
Stain Intensity (AU)
WH
GL
PL
ML
CA3 CB
CA3 NP
B Immunodetection of pGSK3β
C pGSK3β/GSK3β
1.5
1.0
0.5
0
Stain Intensity (AU)
WH
GL
PL
ML
CA3 CB
CA3 NP
Control
Caloric Restricted

Figure S5

Immunodetection of PGC-1α

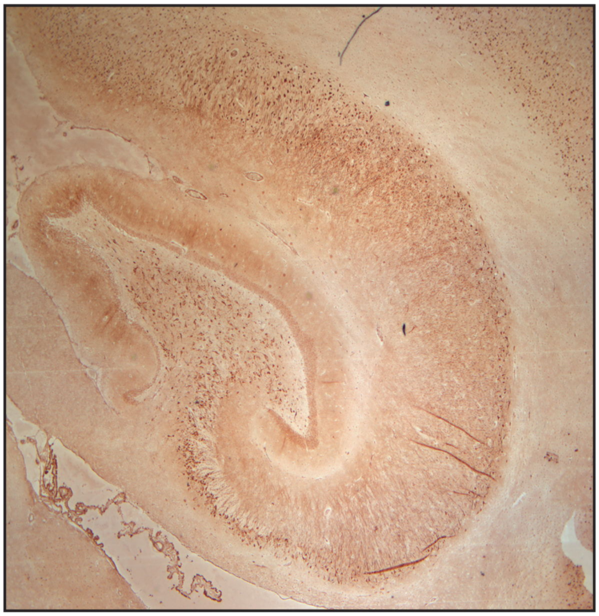

Whole Hippocampus (Monkey)

Figure S6

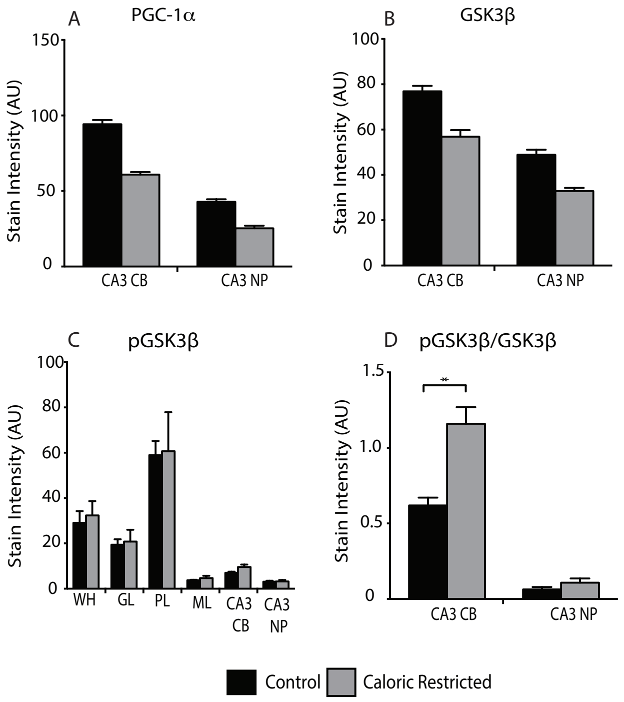

A
PGC-1α
Stain Intensity (AU)
150
100
50
0
CA3 CB
CA3 NP
B
GSK3β
Stain Intensity (AU)
100
80
60
40
20
0
CA3 CB
CA3 NP
C
pGSK3β
Stain Intensity (AU)
100
80
60
40
20
0
WH
GL
PL
ML
CA3 CB
CA3 NP
D
pGSK3β/GSK3β
Stain Intensity (AU)
1.5
1.0
0.5
0
*
CA3 CB
CA3 NP
Control
Caloric Restricted
